# New SARS-CoV-2 Omicron Variant with Spike Protein Mutation Y451H, Kilifi, Kenya, March–May 2023

**DOI:** 10.3201/eid2911.230894

**Published:** 2023-11

**Authors:** Mike J. Mwanga, Arnold W. Lambisia, John Mwita Morobe, Nickson Murunga, Edidah Moraa, Leonard Ndwiga, Robinson Cheruiyot, Jennifer Musyoki, Martin Mutunga, Laura M. Guzman-Rincon, Charles Sande, Joseph Mwangangi, Philip Bejon, Lynette Isabella Ochola-Oyier, D. James Nokes, Charles N. Agoti, Joyce Nyiro, George Githinji

**Affiliations:** Kenya Medical Research Institute Wellcome Trust Research Programme, Kilifi, Kenya (M.J. Mwanga, A.W. Lambisia, J.M. Morobe, N. Murunga, E. Moraa, L. Ndwiga, R. Cheruiyot, J. Musyoki, M. Mutanga, C. Sande, J. Mwangangi, P. Bejon, L.I. Ochola-Oyier, D.J. Nokes, C.N. Agoti, J. Nyiro, G. Githinji);; University of Warwick, Coventry, UK (L.M. Guzman-Rincon, D.J. Nokes);; Pwani University, Kilifi (C.N. Agoti, G. Githinji)

**Keywords:** COVID-19, Omicron, respiratory infections, severe acute respiratory syndrome coronavirus 2, SARS-CoV-2, SARS, coronavirus disease, zoonoses, viruses, coronavirus, FY.4, Kilifi, Kenya

## Abstract

We report a newly emerged SARS-CoV-2 Omicron subvariant FY.4 that has mutations Y451H in spike and P42L in open reading frame 3a proteins. FY.4 emergence coincided with increased SARS-CoV-2 cases in coastal Kenya during April–May 2023. Continued SARS-CoV-2 genomic surveillance is needed to identify new lineages to inform COVID-19 outbreak prevention.

As of August 2023, >340,000 test-confirmed COVID-19 cases and 5,689 COVID-19–related deaths had been reported in Kenya (*1*). Seropositivity rates in rural and urban populations were high, and vaccine uptake was low (≈28% of the adult population received >1 dose) (*2*). By August 2022, a total of 69%–81% of rural (Kilifi and Siaya) and 89%–95% of urban (Nairobi and Kisumu) adults in Kenya had IgG against the SARS-CoV-2 spike protein (*3*).

Genomic surveillance is critical to determine origins of new waves, evolution, and spread patterns of SARS-CoV-2. As of June 2023, a total of 7 distinct waves of SARS-COV-2 infections have been observed in Kenya (*1*; G. Githinji et al., unpub. data, https://doi.org/10.1101/2022.10.26.22281446). The last 3 waves were dominated by Omicron BA.1-like, BA.5-like, and BQ-like lineages (in that order), associated with increases in SARS-CoV-2 cases because of virus mutations that conferred transmission advantage or escape from preexisting immunity (*4*).

In coastal Kenya, the Kenya Medical Research Institute Wellcome Trust Research Programme is conducting SARS-CoV-2 genomic surveillance across 5 health facilities within the Kilifi Health and Demographic Surveillance System (KHDSS) (*5*). Up to 75 respiratory samples are collected weekly from persons of all ages with acute respiratory illness at participating health facilities. SARS-CoV-2 reverse transcription PCR and sequencing are performed on virus-positive samples from KHDSS health facilities and other collaborating health facilities across Kenya.

SARS-CoV-2 positivity rates in KHDSS health facilities increased from 1.2% during the week beginning March 27, 2023, to 42.9% during the week beginning April 24, 2023 (Figure, panel A). After dropping to 23.5% during the first week of May, rates remained at 5.0%–7.7% over the next 3 weeks. 

**Figure:**
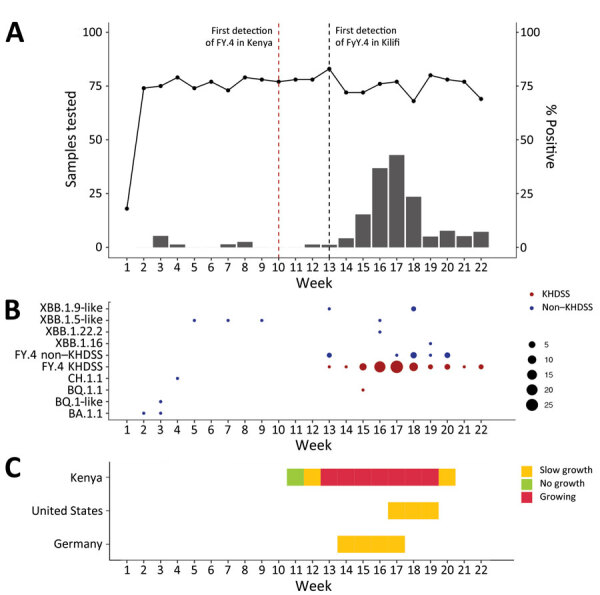
Number of positive samples, distribution, and growth rate for new SARS-CoV-2 Omicron variant with spike protein mutation Y451H, Kilifi, Kenya, March–May 2023. The x axes indicate calendar weeks beginning on January 1, 2023. A) Weekly number of collected samples (black data line) and positive SARS-CoV-2 cases (data bars) in health facilities within the KHDSS during January–May 2023. Vertical dotted lines indicate the weeks when the FY.4 lineage was first detected in Kenya (red) and Kilifi (black). B) Weekly distribution of SARS-CoV-2 lineages observed in samples processed at the Kenya Medical Research Institute Wellcome Trust Research Programme from KHDSS (red dots) and non-KHDSS (blue dots) health facilities during January–May 2023. C) Growth rate estimates for the Omicron FY.4 variant in Kenya relative to those in the United States and Germany. KHDSS, Kilifi Health Demographic Surveillance System.

During January–May, a total of 120 (7.4%) of 1,612 samples collected from KHDSS health facilities were positive for SARS-CoV-2. We sequenced 96 (80%) of 120 samples that had PCR cycle threshold values <35 by using GridION (Oxford Nanopore Technologies, https://www.nanoporetech.com) (n = 35) or MiSeq (Illumina, https://www.illumina.com) (n = 61) instruments; we recovered 76 (79%) genomes with >70% coverage. We also received 39 SARS-CoV-2–positive samples from health facilities outside the KHDSS and sequenced 32 (82%) of them, yielding 25 (78%) genomes (Appendix Table).

We assigned the 76 genomes from KHDSS health facilities to 2 lineages: BQ.1.1 (n = 1) and FY.4 (n = 75). The SARS-CoV-2 infection rate increase observed in late March coincided with detection of the new Omicron FY.4 lineage (Figure, panel B). In Kenya, FY.4 was first observed in Lamu County (n = 6) on March 10, 2023 (Figure, panel A); in KHDSS, FY.4 was first identified from samples collected on March 27 and, in April and May, it became the dominant lineage, representing 98% of all SARS-CoV-2 cases. By May 31, according to the GISAID database (https://www.gisaid.org), FY.4 had been detected in 4 other counties: Mombasa (n = 2), Narok (n = 2), Nairobi (n = 7), and Kiambu (n = 31). The FY.4 subvariant has since been detected in Austria, Belgium, Germany, Italy, India, Sweden, Canada, France, China, Australia, Spain, the United Kingdom, and the United States (*6*).

In KHDSS health facilities, patients infected with FY.4 had cough (98%), fever (78%), and nasal discharge (74%); 7% had difficulty breathing (Table). Only 13 (16%) participants reported receiving >1 dose of a COVID-19 vaccine. A serosurveillance study (February–June 2022) found that 67% of unvaccinated KHDSS health facility residents had SARS-CoV-2 IgG, indicating a high percentage of this population might have been naturally infected (*3*).

**Table:** Clinical signs and symptoms for patients infected with SARS-CoV-2 Omicron FY.4 variant with spike protein mutation Y451H, Kilifi Health Demographic Surveillance System, Kilifi, Kenya, January–May 2023*

Signs/symptoms	No. (%) patients
Fever	
Yes	57 (78.1)
No	16 (21.9)
Cough	
Yes	72 (98.6)
No	1 (1.4)
Nasal discharge	
Yes	54 (74.0)
No	19 (26.0)
Difficulty breathing	
Yes	5 (6.8)
No	68 (93.2)
Sore throat	
Yes	28 (38.4)
No	45 (61.6)
Body malaise	
Yes	25 (34.2)
No	48 (65.8)
Conscious level	
Alert	73 (100.0)
COVID-19 vaccination status	
Yes	12 (16.4)
No	60 (82.2)
No data	1 (1.4)
COVID19 vaccine doses	
1	3 (4.1)
2	7 (9.6)
No data	63 (86.3)

*Total number of patients was 73.

Compared with other Omicron lineages, FY.4 had 2 amino acid mutations, Y451H in the spike protein and P42L in the open reading frame (ORF) 3a protein. The effect of the Y451H change is unknown; however, an L452R mutation in the receptor-binding domain of the spike protein (near the Y451H site) increased virus infectivity and fusogenicity by enhancing spike stability and cleavage (*7*). Changes within ORF3a epitopes can cause complete loss of CD8+ T-cell recognition of ancestral SARS-CoV-2 lineages and Alpha variant (*8*).

We applied a Bayesian hierarchical model (*9*) to estimate the growth rate of FY.4-like lineages in Kenya. Growth estimates serve as an epidemic warning system for lineages that have consistent increases in frequency for >2 consecutive weeks. We compared growth rate estimates from Kenya with those from Germany and the United States, the only other countries reporting FY.4 cases for >2 consecutive weeks during the last weeks of May 2023 (Figure, panel C). The model warned of a high epidemic potential in Kenya from March 26 through May, suggesting continued increases in cases attributed to the FY.4 lineage.

In conclusion, we detected emergence of a new Omicron lineage with unique spike and ORF3a gene mutations in coastal Kenya by using SARS-CoV-2 genomic surveillance. FY.4 subvariant detection coincided with an increase in SARS-CoV-2 cases in Kilifi. FY.4 was also detected in other parts of Kenya, and growth estimates suggest potential for continued spread of the FY.4 subvariant. Continued SARS-CoV-2 genomic surveillance is critical for identifying new lineages to inform COVID-19 prevention measures.

**Appendix.** Additional information for new SARS-CoV-2 Omicron variant with spike protein mutation Y451H, Kilifi, Kenya, March–May 2023.
