# Supplementary material for: New SARS-CoV-2 Omicron Variant with Spike Protein Mutation Y451H, Kilifi, Kenya, March–May 2023
Source: Emerg Infect Dis. 2023 Nov;29(11):2376–9. doi: 10.3201/eid2911.230894 (PMC10617346; doi:10.3201/eid2911.230894)
Supplement: Supplementary file 1 — Appendix. Additional information for new SARS-CoV-2 Omicron variant with spike protein mutation Y451H, Kilifi, Kenya, March–May 2023. [file 23-0894-Techapp-s1.pdf]

Article DOI: https://doi.org/10.3201/eid2911.230894

EID cannot ensure accessibility for supplementary materials supplied by authors. Readers who have difficulty accessing supplementary content should contact the authors for assistance.

New SARS-CoV-2 Omicron Variant with Spike Protein Mutation Y451H, Kilifi, Kenya, March–May 2023

Appendix

Data Availability

GISAID Identifier: EPI_SET_230627zw, https://doi.org/10.55876/gis8.230627zw

All genome sequences and associated metadata in this dataset are published in the GISAID EpiCoV database. Contributors of each individual sequence and details, such as accession number, virus name, collection date, originating and submitting laboratories, and author list, are available at https://doi.org/10.55876/gis8.230627zw.

Data Snapshot

EPI_SET_230627zw is composed of 101 individual genome sequences. The collection dates range from March 27 to May 31, 2023. Data were collected in 1 country and territory. All sequences in this dataset were compared with hCoV-19/Wuhan/WIV04/2019 (WIV04), the official reference sequence used by GISAID (EPI_ISL_402124) (https://gisaid.org/WIV04).

Appendix Table. Summary showing numbers of collected samples, SARS-CoV-2 positive samples, and those sequenced at the Kenya Medical Research Institute Wellcome Trust Research Programme during January–May 2023*

Characteristics	KHDSS	Non-KHDSS
No. samples collected for testing†	1612	0
No. SARS-CoV-2–positive samples	120	39
No. sequenced samples‡	96	32
No. sequences with >70% coverage	76	25
No. subvariants by county	Kilifi (FY.4, n = 75; BQ.1, n = 1)	Kilifi (BA.1.1, n = 2); Kwale (FY.4, n = 2); Kiambu (FY.4, n = 5); Mombasa (XBB.1.5-like, n = 1); Nairobi (BQ.1.1, n = 1; CH.1.1, n = 1; XBB.1.5-like, n = 4; XBB.1.9-like, n = 3; XBB.1.16, n = 1; XBB.1.22.2, n = 1; FY.4, n = 4)

*The KHDSS column represents samples collected from health facilities within the mapped Kilifi Health Demographic Surveillance System area. The Non-KHDSS column represents positive samples that were previously tested and collected outside the KHDSS area and sequenced at Kenya Medical Research Institute Wellcome Trust Research Programme. KHDSS, Kilifi Health Demographic Surveillance System.
†No samples were shared for testing from non-KHDSS health facilities. We only received previously tested samples.
‡Samples were sequenced if they had a PCR cycle threshold value <35 and nucleic acid concentration >18 ng/µL after the PCR cleanup step.
